# Examining the Twitter Discourse on Dementia During Alzheimer’s Awareness Month in Canada: Infodemiology Study

**DOI:** 10.2196/40049

**Published:** 2022-10-26

**Authors:** Juanita-Dawne Bacsu, Allison Cammer, Soheila Ahmadi, Mehrnoosh Azizi, Karl S Grewal, Shoshana Green, Rory Gowda-Sookochoff, Corinne Berger, Sheida Knight, Raymond J Spiteri, Megan E O'Connell

**Affiliations:** 1 Department of Psychology Canadian Centre for Health and Safety in Agriculture University of Saskatchewan Saskatoon, SK Canada; 2 College of Pharmacy and Nutrition University of Saskatchewan Saskatoon, SK Canada; 3 Department of Computer Science University of Saskatchewan Saskatoon, SK Canada; 4 Department of Psychology University of Saskatchewan Saskatoon, SK Canada

**Keywords:** Twitter, social media, dementia, Alzheimer disease, awareness, public health campaigns

## Abstract

**Background:**

Twitter has become a primary platform for public health campaigns, ranging from mental health awareness week to diabetes awareness month. However, there is a paucity of knowledge about how Twitter is being used during health campaigns, especially for Alzheimer’s Awareness Month.

**Objective:**

The purpose of our study was to examine dementia discourse during Canada’s Alzheimer’s Awareness Month in January to inform future awareness campaigns.

**Methods:**

We collected 1289 relevant tweets using the Twint application in Python from January 1 to January 31, 2022. Thematic analysis was used to analyze the data.

**Results:**

Guided by our analysis, 4 primary themes were identified: dementia education and advocacy, fundraising and promotion, experiences of dementia, and opportunities for future actions.

**Conclusions:**

Although our study identified many educational, promotional, and fundraising tweets to support dementia awareness, we also found numerous tweets with cursory messaging (ie, simply referencing January as Alzheimer’s Awareness Month in Canada). While these tweets promoted general awareness, they also highlight an opportunity for targeted educational content to counter stigmatizing messages and misinformation about dementia. In addition, awareness strategies partnering with diverse stakeholders (such as celebrities, social media influencers, and people living with dementia and their care partners) may play a pivotal role in fostering dementia dialogue and education. Further research is needed to develop, implement, and evaluate dementia awareness strategies on Twitter. Increased knowledge, partnerships, and research are essential to enhancing dementia awareness during Canada’s Alzheimer’s Awareness Month and beyond.

## Introduction

Dementia is a rapidly growing public health challenge. In Canada, over 402,000 people live with dementia, and this number is projected to increase as the population ages [1]. Dementia is a general term used to describe a group of diseases and conditions affecting the brain, ranging from Alzheimer disease to Parkinson disease [2]. Alzheimer disease is the most common cause of dementia and accounts for approximately 64% of all dementias in Canada [3].

Stigma (eg, negative beliefs, labels, and discriminatory actions) is a significant issue experienced by people living with dementia and their care partners [4-6]. Almost 50% of Canadians would not want others to know if they had dementia [7]. Inadequate knowledge about dementia and lack of awareness contribute to stereotypes and discrimination against people living with dementia [8-10].

Stigma of dementia can lead to feelings of depression, anxiety, social isolation, and a decreased quality of life for people with dementia and their care partners [10-12]. Moreover, stigma of dementia can deter people from accessing educational information, health services, and supports, thus creating a barrier to timely diagnosis of dementia [7]. A timely diagnosis enables people with dementia to access support services, plan for the future, and access interventions that may improve their quality of life [10]. Consequently, dementia awareness campaigns are vital to reducing stigmatic beliefs, supporting cognitive health promotion, and optimizing timely diagnosis.

Globally, there are increasing efforts to increase awareness of dementia through various Alzheimer’s Awareness Months [12]. For example, the United States has its Alzheimer’s Awareness Month in November, the World’s Alzheimer’s Awareness month is in September, and Canada has its Alzheimer’s Awareness Month in January. In the past, these awareness campaigns have targeted traditional media such as the radio, television, and print media (eg, magazines, newspapers, and newsletters). More recently, social media websites (such as Facebook, Instagram, and Twitter) are being used as primary venues for hosting awareness campaigns.

With a daily average of 500 million tweets, Twitter is becoming a powerful platform for public health campaigns ranging from mental health awareness [13] to breast cancer awareness month [14]. However, no studies have examined Twitter campaigns for Alzheimer’s Awareness Month. Accordingly, the purpose of our study was to examine the Twitter discourse on dementia during Alzheimer’s Awareness Month in Canada.

## Methods

### Ethical Considerations

Compared to traditional research with human participants, tweets posted publicly on Twitter can be used for research without additional consent or ethics approval [13,15,16]. Consequently, institutional ethics approval was not obtained as our data collection focused on tweets shared within the public domain. Nevertheless, any personal identifying information (such as @handles, usernames, and URLs) was removed to ensure anonymity and protect the identity of the Twitter users.

### Recruitment

**Figure 1:**
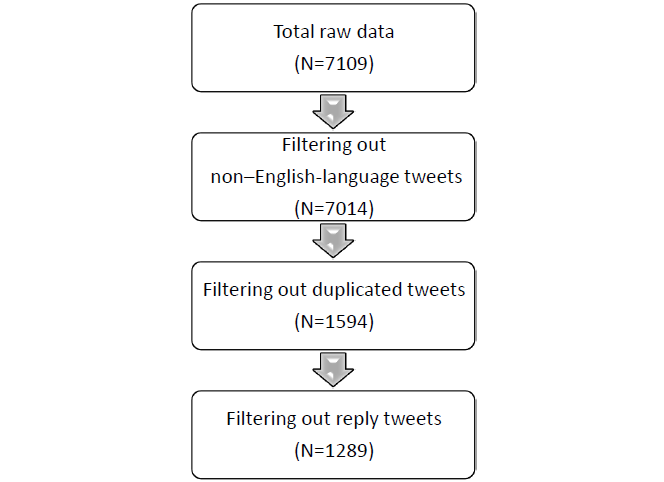
Tweet filtering process.

Tweets were collected using Twint, an advanced scraping tool written in Python. Twint allows users to scrape tweets without the use of Twitter’s application programming interface, thus enabling it to avoid certain restrictions such as the number of tweets scraped, the frequency and time period of scrapes, and the requirement of a Twitter account [17]. Our study focused on Canada’s Alzheimer’s Awareness Month; consequently, we used Twint to asynchronously scrape tweets for the time frame of January 1 to January 30, 2022. Our search terms consisted of various phrases of Alzheimer’s Awareness Month (ie, “#AlzheimersAwarenessMonth,” “#AlzheimersAwareness,” “#AlzAwareness,” “#dementiawareness,” “dementia month,” “dementia awareness month,” “dementia awareness,” “Alzheimer’s awareness month,” “Alzheimer’s awareness,” “Alzheimer's month,” and “January is Alzheimer’s Awareness month”) or tweets using a combination of either “Canada” and “dementia” or “Alzheimer’s.” We also searched for tweets posted from Canada’s national and provincial Alzheimer organizations (ie, @alzCanada, @AlzheimerOnt, @AlzheimerSK, @DementiaAB_NT @AlzheimerNS, @AlzheimerNB, @AlzheimerPEI, @alzheimerMB, @AlzheimerBC, @asnl2, and @FqsaAlzh). Given that our search focused on Canada’s Alzheimer’s Awareness Month, we only included tweets from Canada. Our initial search resulted in a total of 7109 tweets. We used filters to exclude non–English-language tweets, reply tweets, duplicate tweets, and spam tweets, resulting in 1289 tweets (see Figure 1). The remaining 1289 tweets were extracted to an Excel (Microsoft Inc) spreadsheet for thematic analysis.

It is important to note that our total number of relevant tweets (n=1289) is comparable to other Twitter awareness studies. For example, in Makita et al’s [13] study on the mental health discourse on Twitter during a mental health awareness campaign, the authors analyzed 1200 tweets. In addition, Chung [14] analyzed 1018 tweets to examine the discourse during breast cancer awareness month.

### Data Analysis

**Figure 2:**
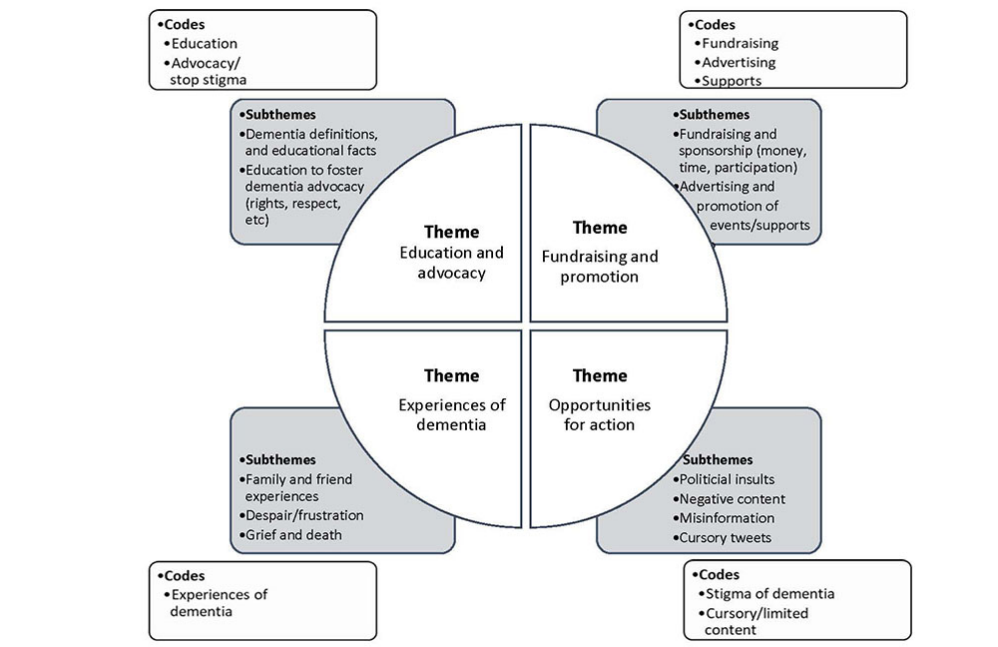
Thematic Map.

Tweets were analyzed using a 5-stage inductive thematic analysis process [18]. First, the researchers (JB, MEO, and RJS) immersed themselves in the by-reading and rereading of 150 tweets to support data familiarization of the Tweets. We used memos to capture any preliminary ideas about the data (eg, similarities, differences, and interesting points) to assist in the development of our initial codes [19]. Second, an open coding approach was used by rereading the initial 150 tweets and developing a code for each tweet, which led to a list of codes. Third, we created a codebook to identify each code name, code definition, and specific examples of relevant tweets for each code. We completed pilot tests with our codebook to help support consistency and intercoder reliability during the coding process. For example, our codebook was pilot tested by having our full team independently code the same set of data (eg, 75 tweets), and then compare coding to discuss coding similarities, differences, or uncertainties. Following our pilot test, tweaks were made to ensure a robust codebook (eg, adding inclusion and exclusion criteria and providing additional examples for each code) to help improve the clarity of the codes. The final codebook consisted of 8 codes, including the following: fundraising (eg, “please donate to Alzheimer’s research”), advocacy or stopping the stigma on dementia (eg, “let’s break the stigma of dementia…”), cursory or limited content (eg, “January is Alzheimer’s Awareness Month”), advertising (eg, “attend our conference on…”), experiences with dementia (eg, “Mom died of Alzheimer’s…”), advocacy or stopping the stigma on dementia (eg, “…the only truly deranged are the ones having their Alzheimer's medications filled…”), supports (eg, “each month we offer a caregiver support group…”), and education (eg, “dementia describes a group of symptoms, such as memory loss…”). Each tweet was coded independently by 2 coders to support intercoder reliability. All of our coders were experienced in conducting thematic analysis and have had previous experience in coding tweets [20]. Any coding uncertainties or disputes were resolved through discussion and consensus with 2 coders. However, if consensus could not be reached, the first author acted as a third reviewer to resolve any coding discrepancies. Fourth, once the coding was completed, team meetings were held to discuss relationships and theme development among the codes. More specifically, we used Braun and Clark’s [18] proposed theme generation questions to help support our theme development process. For example, we discussed the coherence of our themes, whether our data supported our themes, and whether we were missing any themes. Fifth, we used a thematic map to document our process for grouping our codes into subthemes that supported the development of our 4 main themes (see Figure 2).

### Trustworthiness and Rigor

We used 4 measures to support trustworthiness and rigor in our research. First, multiple coders (eg, each tweet was coded independently by 2 researchers) were used to help ensure a rich data analysis by reducing the potential for individual bias and subjectivity during our coding process [21]. Second, we calculated intercoder reliability scores to assess coding agreement between our different pairs of researchers who were coding the same sets of data. More specifically, our intercoder reliability rate was determined by calculating the percentage of coding agreement among our 6 pairs of coders to establish our overall group average, which was 88.83% (see Table 1 for a breakdown of our intercoder reliability scores). Third, memo writing was integrated into the data analysis process to enable iterative descriptions to support theme development to be discussed and reviewed by the full research team [19]. For example, researchers used analytic memos to document interesting aspects of the data and emerging impressions of relationships and patterns (eg, similarities and differences) to help inform the development of overarching themes [22]. Illustrative tweets of each theme were also identified collaboratively by the team during the memo writing process. Fourth, interdisciplinary reflexivity (eg, reflecting on each researchers’ identity, assumptions, roles, theoretical underpinnings, and positioning) was used to help inform and avoid any potential areas for bias [23]. Team members were representative of different disciplines and academic backgrounds such as psychology, nutrition, community health and epidemiology, and computer science. Moreover, our coding pairs (ie, 2 coders analyzing each tweet) were purposefully selected to ensure researchers from different disciplines were partnered together to provide a more nuanced analysis. Drawing on this interdisciplinary lens (researchers with different disciplines, training, and expertise) enabled a more comprehensive and in-depth interpretation of the data than could have been achieved by a single discipline [23].

**Table 1:** Intercoder reliability.

Coder pairs	Intercoder agreement, %
JDB and CB	84.00
KSG and MA	80.00
JDB and SA	86.00
AC and RGS	91.00
MEO and RS	98.00
SG and SK	94.00
Intercoder reliability average	88.83

## Results

Based on our analysis, 4 themes were identified on Twitter during Alzheimer’s Awareness Month in Canada: (1) dementia education and advocacy, (2) fundraising and promotion, (3) experiences of dementia, and (4) opportunities for future actions.

### Dementia Education and Advocacy

A predominant theme identified in the tweets was dementia education and advocacy. Several tweets shared facts and statistics about dementia in Canada. For example, tweets often provided definitions and descriptions of dementia. Numerous tweets provided dementia-related statistics, such as the number of Canadians living with dementia. Some tweets discussed statistics of dementia care partners in addition to highlighting brief facts (eg, early diagnosis benefits, risk factors, etc). These educational tweets are illustrated in the examples below.

Dementia is used to describe a group of symptoms, such as memory loss, confusion, mood changes and communication difficulties. Alzheimers disease is the most common cause of dementia…

January is Alzheimer Awareness Month. #DYK that more than 500,000 people are currently living with dementia today in Canada? That number is expected to rise to 912,000 by 2030…

January is #AlzheimersAwarenessMonth, an opportunity to learn more about dementia. 1 in 5 Canadians have experienced caring for someone with dementia. Early diagnosis can lead to a better quality of life.

Many tweets focused on advocacy related to stopping stigma and recognizing the rights of people living with dementia. For example, tweets on stopping stigma described the need to combat or end stigma against people living with dementia. These tweets often had a strong emphasis on the need for more education and dialogue on dementia to address misconceptions and false information. Tweets on stopping the stigma on dementia are captured in the following examples.

January is #AlzheimersAwarenessMonth. Lets end the stigma surrounding dementia by talking about it, educating ourselves, and supporting those who live with dementia and care partners. Learn from our variety of resources…

…We encourage you to learn more about Alzheimer's disease throughout the month and help us smash the stigma surrounding it… get started by learning about these common myths surrounding the disease…

Several tweets discussed respecting the rights of people with dementia. For example, family care partners often emphasized the need to recognize the rights and dignity of people living with dementia. Advocacy tweets highlighting the need to respect people with dementia are illustrated in the examples below.

January marks #AlzheimersAwarenessMonth. People living with dementia deserve to have their rights respected and protected, including the right to manage their own lives. Learn more about the rights for people living with dementia…

January is #AlzheimersAwarenessMonth I advocate to ensure my beloved Mum receives the care, dignity, and respect she deserves while living with dementia so much more to do!!

### Fundraising and Promotion

Many tweets identified fundraising and promotional efforts to support dementia awareness during Alzheimer’s Awareness Month in Canada. More specifically, fundraising frequently involved requests for money, donation of time, or participation in an event (eg, walk, run, bike, skate, etc), often with a focus of supporting people with dementia and fighting for better outcomes. The following examples illustrate fundraising tweets.

We need your help to find a cure! Please donate to Alzheimer's today! #Alzheimers #AlzheimersResearch #dementia #alzheimersawareness #caregiver #dementiacare #dementiaawareness #seniorcare #aging #alzheimersdisease #mentalhealth

On Saturday, February 5, 2022, starting at 12:00am (midnight), Steve McNeil, a mailman and recreational hockey referee from Etobicoke, ON is coming to Windsor to skate for 19 hours and 26 minutes to raise awareness and funds for Alzheimers disease…

Day 12, 1317 press ups completed, 705 to go. Raising funds for Alzheimers Society and Alzheimer's awareness. Donate via the link…

Promotional efforts did not involve requests for any type of fundraising but focused on drawing the public’s attention toward an array of dementia-related efforts. These promotions took 2 forms. The first form of tweets promoted a range of opportunities to connect people to knowledge and education about dementia, including research, conferences, webinars, books, and videos. These promotional tweets focused on educational events are highlighted in the examples below.

SAVE THE DATE! Our 10th Annual Alzheimers Awareness Conference will take place virtually on Thursday, January 27th from 9am-12:30pm. Stay tuned for further registration announcements…

It's #AlzheimersAwarenessMonth! Register for the @ccna_ccnv's event… to learn more about supporting the well-being of care partners for persons living with #dementia. Sign up here…

The second type of promotional tweets amplified social supports for people living with dementia and their care partners. Tweets highlighting social supports (eg, support groups and telephone helplines) are demonstrated in the following examples.

It's Alzheimer's Awareness Month. We know being a caregiver for a loved one can be challenging. Call the 2- 1-1 helpline to find supports in your community from home assessments to respite supports, Alzheimer’s programs and more.   Dial 2-1-1…

Are you a caregiver for someone with dementia? We have a support group for you. Join us the first Thursday of each month…

### Experiences of Dementia

A third theme within the data described experiences related to knowing someone with dementia. Notably absent from our findings were tweets sharing the lived experiences and the direct perspectives of people living with dementia. Rather, tweets tended to focus on the experiences of family members and friends of people with dementia. Within this theme, experiences of dementia were shared in concert with the awareness month campaign to bolster the campaign, but other experience-based tweets appeared disconnected from the campaign. Appreciation for the lived experience of dementia was also heralded as an important component of Alzheimer’s Awareness Month.

Today marks one year without my uncle [sic] & Ive been raving about getting a purple butterfly for him for Alzheimers awareness…

January is Alzheimer's Awareness Month! Let's understand the experiences of people living with #dementia to impact their lives positively…

Although some tweets highlighted the awareness month campaign by sharing a personal story, many tweets appeared to be distinct and not connected to the advocacy campaign. Instead, these appeared to represent the regular and constant flow of personal dementia experiences shared via Twitter [24].

Just over a month ago I moved back home to be with mum as shed received a diagnosis of Alzheimers. Im sharing this with you now as I know a few of you have been in my shoes with your own parents and Ive realised Id appreciate support & advice on this journey.

My dad has dementia and cant be alone. Every month my mom goes to dinner w/friends. Comes home and asks why he hasnt gone to bed. Every month I say because he is waiting for you. I cant decide if she thinks shes being cute. Because I already know shes got a selfish streak.

My grandpa has dementia and Im the only one in the family who visits him weekly and helps him. I asked my family to visit him at least once a month in our group text. One person said theyd visit this week. I hope he haunts them when he dies.

Tweets expressing grief and loss were notable among these personal experiences and frustration with inadequate care at the end of life for people with dementia. Although some positive experiences with dementia were shared, many tweets featured negative experiences including despair, frustration, and issues of grief. Among these tweets, there was a predominant focus on death and the end-of-life stage among people with dementia.

And there's the call from mom. Grandma passed away peacefully this morning after over a decade fighting alzheimer's. It was very expected, she's been in steep decline over the last month. I lost my grandma years and years ago, but it still hurts to lose her again.

Mom died of Alzheimers, yesterday. Last month, owing to a fall, she was hospitalized. They were overwhelmed with Covid patients, so there were no beds. She was strapped into a gurney, in a hallway, confused and alone…

### Opportunities for Action

Although numerous tweets offered educational content, several tweets lacked informative content. For example, these tweets usually made cursory references to January as Alzheimer’s Awareness Month without providing any educational information or additional context. Examples of cursory tweets are illustrated below.

Did you know that January is #Alzheimer Awareness Month in Canada?

January is Alzheimers Awareness Month!

January is Canadas Alzheimer Awareness Month #Alzheimers #alzheimersawarenessmonth #Canada #dementia #findacure #cure #memory #memoryloss…

While these tweets promoted the campaign and increased general awareness, they also indicate an opportunity for increasing informative content to counter stigma (eg, negative attitudes, beliefs, and discriminatory behavior) of dementia [25,26]. For example, many tweets used stigmatizing language such as “suffer” and “endure” to describe people living with dementia. Moreover, several tweets made derogatory comments and political ridicule about dementia. Some tweets also shared false information about the COVID-19 vaccine causing dementia. These tweets are documented in the examples below.

Checking out at the liquor store. Guy at register:how old are you? Me: *genuinely trying to remember my age* dahhhh......twentyyyyy-nine? *Realizing how stupid I am* I turn 30 next month. The dementia's settling in way earlier than I thought.

You know how some politicians are documented as saying one thing then contradicting it, within a month to a year... Shouldn't people check if they have dementia, instead of accusing of lies/being 'evil puppets' ect ect.

These people aren't dumb, they know exactly what they're doing. They're counting on you just writing them off as stupid. The only truly deranged are the ones having their Alzheimer's medications filled every month. But thats an entirely different conversation.

Alzheimers is a vax side-effect. Perhaps they are slowly introducing their advances in breakthrough treatments to what they have injected us with…

These negative comments and misinformation only further increase the stigma against people living with dementia. Recent reports indicate that negative attitudes and misinformation can act as a barrier for people with dementia in seeking a timely dementia diagnosis [8,10]. Consequently, these examples highlight opportunities for future Twitter campaigns to provide targeted tweets to counter stigmatizing language and misinformation.

## Discussion

### Principal Findings

Our study aimed to examine the Twitter discourse on dementia during Alzheimer’s Awareness Month in Canada. Understanding Twitter content during Alzheimer’s Awareness Month is vital to informing targeted education and messaging strategies. A growing body of literature suggests that Twitter may provide an effective means to educate audiences on public health issues, raise awareness, and counter misinformation on stigmatized diseases such as dementia [13,27,28]. Using thematic analysis, we found 4 main themes during Alzheimer’s Awareness Month ranging from fundraising to opportunities for future actions. 

Our study identified many educational, advocacy, promotional, and fundraising tweets to support people living with dementia. Although several tweets provided informative content, we also identified numerous tweets that contained cursory messaging by simply referencing January as Canada’s Alzheimer’s Awareness Month. While these tweets promoted the campaign and increased general awareness, they also indicate an opportunity for targeting educational content to counter misinformation and correct stigmatizing language. Many tweets used a negative discourse and stigmatizing words such as “suffer” and “endure” to describe people living with dementia. However, stigmatizing language perpetuates misinformation and myths by implying that people with dementia are not able to live meaningful lives. Moreover, negative discourse against people living with dementia can have important public health implications. How society communicates about dementia influences how policy makers, practitioners, and the general public value the lives of people with dementia. Studies show that stigma can lead to inequitable access to health care services for people living with dementia [29-31]. Consequently, targeted educational campaigns are needed to address stigmatizing language and misinformation about dementia.

Existing literature on mental health and cancer show that educational awareness campaigns can have positive outcomes at both the societal and individual levels [31-33]. For example, educational campaigns can counter public stigma at the societal level and alleviate self-stigma to improve self-confidence at the individual level [31]. However, a paucity of knowledge exists on educational campaign strategies to address the stigma on dementia, especially on social media platforms such as Twitter [34,35]. Future research is necessary to develop and evaluate educational campaign strategies on social media to counter stigma and correct stigmatizing language used against dementia.

Similar to existing studies [36,37], our study on Twitter discourse found that many tweets shared experiences of dementia. More specifically, people shared personal narratives related to issues of grief, bereavement, and death of family members with dementia. Among these tweets, there was a notable focus on death and the end-of-life stage of dementia. Although dementia due to all etiologies is incurable (ie, no disease-modifying intervention currently exists; rather, symptom management and risk reduction remain the primary interventions), the public is often unaware that dementia does not progress in a linear fashion and varies from person to person [38,39]. Tweets focused on negative aspects of the end-of-life stage may enhance the awareness of support needs during this stage, but they may also exacerbate existing stereotypes against dementia focused on frailty, suffering, and one’s inability to live well with dementia [20]. Conversely, tweets sharing real-life stories of people living well with dementia may help to counteract negative stereotypes of people with dementia.

Our study found that the lived experiences and perspectives of people living with dementia were notably absent from the tweets. Research suggests that sharing diverse lived experiences of dementia can help reduce the stigma on dementia by fostering knowledge, understanding, and empowerment [38]. Accordingly, future Alzheimer awareness campaigns on Twitter could be enhanced by highlighting diverse voices and insights of people living with dementia. For example, existing Twitter studies on mental health [13], flu vaccines [40], and breast cancer [14] highlight the beneficial role of engaging social media influencers (eg, celebrities) and champions (eg, advocates, care partners, and people with lived experience) to promote awareness and counter stigmatizing discourse on social media. Accordingly, partnerships with diverse stakeholders (eg, people living with dementia, care partners, celebrities, social media influencers, etc) could play an important role in improving dementia dialogue and awareness on Twitter. Further research is necessary to examine and evaluate awareness campaigns that work in direct partnership with different stakeholders.

### Limitations

Although a rigorous process was undertaken to perform this study, our research has limitations. For example, our research focused only on tweets related to Alzheimer’s Awareness Month in Canada. However, examining Alzheimer’s Awareness Months in other countries may add value to understanding different social media strategies and awareness techniques. Moreover, it would be of interest to address cultural differences in perceptions of the disease and factors that may positively influence public discourse. Further research is necessary to investigate Twitter data based on Alzheimer’s Awareness Months in different countries.

Although we aimed to use inclusive search criteria, it is possible that some relevant tweets related to Alzheimer’s Awareness Month may have been missed. More specifically, because our inclusion criteria focused specifically on English-language tweets, it is possible that relevant tweets in other languages were overlooked. Indeed, Canada is a bilingual country, and tweets in the second official language of French were not analyzed in this study. Additional research examining Twitter content in other languages (such as French) may provide a more nuanced and in-depth understanding of the discourse on dementia during Alzheimer’s Awareness Month in Canada.

Given the nature of Twitter, our study did not collect any sociodemographic information based on the Twitter users. Without this information, it is difficult to make any specific inferences or assumptions based on sociodemographic characteristics such as age, ethnicity, or sex and gender. Interviews, focus groups, or surveys with sociodemographic questionnaires may provide insight on sociodemographic characteristics related to effective messaging campaigns tailored to specific audiences during Alzheimer’s Awareness Months.

### Conclusions

Our study examined the Twitter discourse on dementia during Alzheimer’s Awareness Month in Canada. Understanding Twitter content during public health campaigns is critical to informing targeted messaging and educational strategies. Drawing on thematic analysis, our study identified 4 main themes during Alzheimer’s Awareness Month in Canada: dementia education and advocacy, fundraising and promotion, experiences of dementia, and opportunities for future actions.

While several of the tweets provided informative content, we also identified opportunities for action to enhance future awareness month campaigns. For example, we found numerous tweets that contained cursory messaging by simply referencing January as the Alzheimer’s Awareness Month in Canada. While these tweets promoted the campaign and increased general awareness, they also highlighted an opportunity for targeted educational content to correct stigmatizing language and misinformation about dementia. Moreover, awareness strategies partnering with diverse stakeholders (such as people living with dementia, celebrities, social media influencers, advocates, and care partners) may play a pivotal role in fostering dementia education and awareness on Twitter. Further research is needed to develop and evaluate dementia awareness campaigns on social media platforms such as Twitter. Increased knowledge, partnerships, and research are essential to enhancing dementia awareness during Canada’s Alzheimer’s Awareness Month and beyond.
